# Constraint degree in revision total knee replacement: a registry study on 1432 patients

**DOI:** 10.1007/s12306-023-00790-1

**Published:** 2023-05-26

**Authors:** V. Digennaro, M. Brunello, A. Di Martino, A. Panciera, B. Bordini, B. D. Bulzacki Bogucki, R. Ferri, D. Cecchin, C. Faldini

**Affiliations:** 1 1st Orthopaedic and Traumatologic Clinic, IRCCS Istituto Ortopedico Rizzoli, Via G.B. Pupilli 1, 40136 Bologna, Italy; 2 Department of Biomedical and Neuromotor Science-DIBINEM, University of Bologna, Bologna, Italy; 3 Medical Technology Laboratory, Research Institute Codivilla Putti Rizzoli, Via Barbiano 1/10, 40136 Bologna, Italy

**Keywords:** Total knee arthroplasty, Revision, Constraint, Failure, Outcomes

## Abstract

**Purpose:**

Total knee replacement (TKR) failure represents a hard challenge for knee surgeons. TKR failure can be managed in revision with different constraint, related with soft and bone knee damages. The choice of the right constraint for every failure cause represents a not summarized entity. The purpose of this study is identifying distribution of different constraints in revision TKR (rTKR) for failure cause and the overall survival.

**Methods:**

A registry study based on the Emilia Romagna Register of the Orthopaedic Prosthetic Implants (called RIPO) was performed with a selection of 1432 implants, in the period between 2000 and 2019. Selection implants including primary surgery constraint, failure cause and constraint revision for every patient, and divided for constraint degrees used during procedures (Cruciate Retaining-CR, Posterior Stabilized-PS, Condylar Constrained Knee-CCK, Hinged).

**Results:**

The most common cause of primary TKR failure was aseptic loosening (51,45%), followed by septic loosening (29,12%). Each type of failure was managed with different constraint, the most used was CCK in the most of failure causes, such as to manage aseptic and septic loosening in CR and PS failure. Overall survival of TKA revisions has been calculated at 5 and 10 years for each constraint, with a range of 75.1–90.0% at 5 years and 75.1–87.5% at 10 years.

**Conclusion:**

Constraint degree in rTKR is typically higher than primary, CCK is the most used constraint in revision surgery with an overall survival of 87.5% at 10 years.

## Introduction

In the last decades, worldwide, joint replacement procedures (particularly hip and knee arthroplasty) have seen huge growth both in terms of quality and quantity; consequently, revision procedures have also become more common [3, 11, 17]. Recent studies [20] expect an increase in the demand for knee and hip arthroplasty in the next 20 years, estimated at 284% and 401%, respectively. This will likely lead to an increase in the demand for revision procedures.

Revision total knee replacement (rTKR) is used to treat primary arthroplasty failure, allowing the management of different causes of failure, often through an increase in the constraint degree. rTKR is the only option to manage primary failure while preserving joint functionality, while other procedures such as knee arthrodesis or amputation are much more invasive and severely limit joint functionality [1, 8]. The main causes of total knee replacement (TKR) failure are aseptic and septic loosening [16], both leading to bone and soft tissue damage and lesions, ultimately causing joint instability and articular pain. Other causes of TKR failure reported in the literature are painful total knee arthroplasty, periprosthetic fractures, prosthetic dislocation, mechanical failures, prosthetic wear, and others. The rTKR procedure usually is performed using a higher degree of constraint than primary TKR; nevertheless, other technical aspects also aim at maintaining joint stability and reducing pain, specifically through the accurate management of residual bone stock. With the clear and growing importance of rTKR, it is necessary to find the correct indications concerning the appropriate degree of constraint to use during these procedures. The purpose of the current study is to report the long-term results of a large population of patients that underwent rTKR, focusing specifically on the constraint degree used, by analyzing the follow-up data of a regional joint replacement registry in Italy.

## Materials and methods

A registry-based population study has been conducted by reporting and analyzing data collected by the Emilia Romagna Registry of the Orthopaedic Prosthetic Implants (called RIPO for Registro Implantologia Protesica Ortopedica). Emilia Romagna (ER) is an Italian region with 4.5 million inhabitants and data about hip, knee, and shoulder arthroplasty procedures performed throughout the region are collected in the RIPO Register. Founded in 1990, RIPO has a capture rate of approximately 98% on the implants performed in all orthopedic departments of the region (both private and part of the National Healthcare System or Sistema Sanitario Nazionale), involving a total of sixty-two hospitals. The specific design of this register allows comparisons with other important national registries to be made.

For the study, revision knee arthroplasties performed in the period between 2000 and 2019 were selected. Implant failures were considered and reported up to December 31, 2019. The extraction from the database was made on 03/01/2022. Ethical approval for the study was not necessary because the registry collects data as standard practice on all patients, using a format to protect their identity.

A total of 6480 rTKR were performed in ER during the considered period, including revisions of uni-, bi- or tri- compartmental primary arthroplasties. Procedures performed on patients living outside ER region were excluded, to minimize bias due to loss at follow-up. This is because patients living in other regions, which performed primary surgery in ER but an eventual revision outside would not be identified by the RIPO, and therefore survival data would be biased. Furthermore, we excluded the revisions of unicompartmental knee replacements because of the different challenges and possibilities that this type of revision provides. A total of 1432 rTKR performed in the 19 years were included in the study. The following data were considered: degree of constraint used during primary surgery, failure causes and degree of constraint for revision surgery, and revisions implant survival rates at 5 and 10 years of follow-up.

The population selected has been further divided into four groups based on the constraint degrees used during the primary procedure (Cruciate Retaining-CR, Posterior Stabilized-PS, Condylar Constrained Knee-CCK, Hinged). Our population consisted of 690 CR implants, 708 PS, 24 CCK, and 15 hinged.

## Results

From the analysis of the population emerged that the most common cause of primary TKR failure was aseptic loosening, with 737 patients (51,45%), followed by septic loosening with 417 cases (29,12%), painful knee arthroplasty without loosening in 154 patients (10,75%), periprosthetic fracture in 36 patients (2,51%), dislocation 32 cases (2,23%), mechanical failure in 11 patients (0,76%). The prevalence of each cause is reported in Table 1.

**Table 1:** Causes primary failure, subdivided for each type of constraint

	Aseptic	Septic	Pain	Fracture	Dislocation	Mechanical	Wear	Other	Unknown	Tot.
CR	12	4	3	0	0	0	1	0	1	21
	57,10%	19,00%	14,28%	–	–	–	4,76%	–	4,76%	
PS	165	121	46	7	4	2	0	9	3	357
	46,21%	33,89%	12,88%	1,96%	1,12%	0,56%	–	2,52%	0,84%	
CCK	319	168	57	18	14	4	6	7	6	599
	52,25%	28,04%	9,51%	3,00%	2,34%	0,66%	1,00%	1,16%	1,00%	
HINGED	241	124	48	11	14	5	2	4	6	455
	52,96%	27,25%	10,54%	2,41%	3,07%	1,09%	0,43%	0,87%	1,31%	
Total	737	417	154	36	32	11	9	20	16	1432

The data analysis shows how each type of failure was managed and which type of constraint was used (Table 2). From the match between the constraint degree in primary TKR and rTKR emerged that CR primary failures for aseptic loosening, septic loosening, and periprosthetic fracture were predominantly managed with CCK implants, while painful knee arthroplasty without loosening was managed more frequently with hinged implants. PS TKR primary failures for aseptic loosening, septic loosening, and painful knee arthroplasty without loosening were managed primarily with both CCK and Hinged implants. CCK primary implant failures for aseptic loosening were managed predominantly with CCK implants, for septic loosening both CCK and hinged implants were used, and periprosthetic fracture with hinged implants. Hinged primary implant failures for aseptic and septic loosening were managed with hinged implants.

**Table 2:** Data analysis of the population

Primary Constraint degree	Constraint degree after revision	Aseptic loosening	Septic loosening	Pain	Periprosthetic fracture	Dislocation	Mechanical	Wear	Other	Unknown	Total
CR	CR	12	4	3	–	–	–	1	–	1	21
	PS	82	47	24	1	1	2	–	6	1	164
	CCK	147	76	25	9	11	3	4	4	4	283
	Hinged	129	45	30	1	9	3	1	1	3	222
Tot.		370	172	82	11	21	8	6	11	9	690
PS	PS	83	73	22	6	2	–	–	3	2	191
	CCK	162	85	30	9	3	1	1	3	2	296
	Hinged	108	71	18	8	4	1	1	2	3	216
Tot.		355	229	72	24	9	2	2	8	7	708
CCK	PS	–	1	–	–	–	–	–	–	–	1
	CCK	7	6	1	–	–	–	1	–	–	15
	Hinged	–	5	–	2	1	–	–	–	–	8
Tot.		7	12	1	–	1	–	–	–	–	–
Hinged	PS	–	–	–	–	1	–	–	–	–	1
	CCK	3	1	1	–	–	–	–	–	–	5
	Hinged	4	3	–	–	–	1	–	–	-	9
Tot.		7	4	1	–	–	–	–	–	–	15
Total		739	417	156	37	32	11	9	20	16	1432

Revisions with CR implants were performed in a very small population (21 patients), prevalently to manage aseptic loosening (57,1% of cases) in patients with a CR primary implant. Revisions with PS implants (357 patients) were performed for aseptic loosening (46,21% or 165 patients), septic loosening (33,89% or 121 patients), and painful arthroplasty without loosening (12,88% or 46 patients). PS implants during revision procedures were also used in the case of other conditions (as represented in Table 2) in 25 patients or 7% of the PS revision implant population. Revisions with a CCK constraint (599 patients) were the most common and performed for aseptic loosening (53,25% or 319 patients), septic loosening (27,38% or 164 patients), and painful arthroplasty without loosening (9,52% or 57 patients). Also, in this case, CCK implants were used for rTKR for other causes (as reported in Table 2) in 55 patients, or 9,18% of cases. At last, revisions with a hinged implant (455 patients) were used to manage aseptic loosening (52,97% or 241 patients), septic loosening (27,25% or 124 patients), and painful arthroplasty without loosening (10,55% or 48 patients). The remaining causes of failure treated with hinged rTKR are represented in 9,23% of patients (42).

Furthermore, our study evaluated the cumulative survival of each of the four groups (Table 3). For CR revisions the cumulative survival rate was 75,1% (CI 54,3–88,4) at 5 years remaining stable at 10 years of follow-up. The reported cumulative survival for PS implants used in revision arthroplasty was 80,7% (CI 75,8–84,9) at 5 years and 75,3% (CI 69,2–80,5) at 10 years of follow-up. Concerning revision procedures performed using CCK implants, the cumulative survival rate reported was 90,0 (CI 86,9–92,4) at 5 years and 87.5% (CI 84,0–90,4) at 10 years of follow-up. For revisions with hinged implants, the cumulative survival reported was 87,0% (83,1–90,1) at 5 years and 81,7% (CI 75,7–86.5) at 10 years of follow-up. Cox multivariate analysis showed that the difference in survival rates was not statistically significant for the different types of constraints.

**Table 3:** Overall survival of each constraint

Percentage of survival (confidence interval at 95%)						
	Number	1 year	3 years	5 years	7 years	10 years
CR	26	92,3 (73,9–98,1)	79,5 (59,0–91,2)	75,1 (54,3–88,4)	75,1 (54,3–88,4)	75,1 (54,3–88,4)
PS	357	98,0 (95,9–99,0)	88,6 (84,6–91,6)	80,7 (75,8–84,9)	79,3 (74,2–83,6)	75,3 (69,2–80,5)
CCK	599	97,0 (95,2–98,1)	92,0 (89,3–94,1)	90,0 (86,9–92,4)	88,3 (84,9–91,0)	87,5 (84,0–90,4)
Hinged	455	94,2 (91,5–96,0)	88,8 (85,2–91,6)	87,0 (83,1–90,1)	84,2 (79,5–87,9)	81,7 (75,7–86,5)

## Discussion

Primary TKR failure represents a hard challenge for every knee surgeon, with the correct management and adequate choices to make during revision procedures that are still debated in the available literature. TKR failure modalities are reported in the National Joint Registry 2021[23] as aseptic loosening (33,6%), septic loosening (23,5%), instability (14,5%), painful knee arthroplasty without loosening (12,5%), implant wear (11,6%), implant malalignment (6,1%), and periprosthetic fracture (3,8%). The Swedish Knee registry showed infections like the first failure cause (30%), followed by aseptic loosening (28%), patellofemoral arthritis (16%), instability (14%), wear (3%), and fracture (2%). The Finnish and Norway [24, 25] registries reported similar results. The Australian National Joint Registry [26] showed that in 2021, infection was the first cause of failure (26.4%), followed by loosening (22.7%), instability (9.5%), patellofemoral pain (8.4%), painful knee arthroplasty without loosening (8.1%), and patella erosion (6.3%). Our RIPO results are therefore in-line with those of international registers.

Our study investigated what type of constraint in each type of failure was used for revision procedures as reported in the RIPO registry; our results lead to a similar conclusion as that reached by multiple authors. A Korean study [9] on 42 patients, comparing three degrees of constraint (PS, CCK, Hinged), and a Dutch study by S. Japp [13], comparing the use of two different constraints (CCK and Hinged) in the case of instability, showed good functional outcomes and in both cases, there was no clear superiority of one type constraint over the others. Shen et al. [19] studied the type of constraint (PS, CCK, Hinged) in two revision situations: septic (131 patients) and aseptic loosening (344 patients). Using the AORI (Anderson Orthopaedic Research Institute) classification, this study demonstrated that for aseptic loosening, in an AORI type 1 defect PS implants had better results, while in AORI type 3 defects the CCK performed better than hinged implants. For septic loosening, in AORI type 1 PS implants exceeded the CCK outcomes, in AORI type 2 hinged implants obtained better results, and lastly in AORI type 3 defects CCK had higher scores than hinged implants. Similar results were obtained by Hossain [6] with a retrospective study on 349 rTKRs, over a period of 84 months, where the relationship between clinical outcomes and different types of constraints was evaluated. The causes that led to revision surgery were infection (33%), aseptic loosening (15%), and polyethylene wear (13%). Constraints were distributed in the cohort in 126 PS, 149 CCK, and 74 Hinged. The results showed a better result for AKSS (American Knee Society Score) with PS implants, and particularly concerning the Range of Movement (ROM), PS and Hinged implants obtained better results than CCK. These studies showed clinical and functional differences related to the degree of constraint chosen, but with a comparable distribution. In general, a lower degree of constraint was used to manage aseptic failure, while higher constraints were reserved for more severe clinical conditions with bone loss and insufficiency of soft tissue.

Our registry study also compared the survival of different constraints with an intermediate (5 years) and long-term (10 years) follow-up period; the overall survival rate for rTKR ranged between 75.1 and 90.0% at 5-years and between 75.1 and 87.5% at 10-years of follow-up. The results were in agreement with data reported from other international registries [23]. In particular, the Nation Joint Register (NJR) divided implants into PS and semi-constrained, with 5-year and 10-year follow-up: the 5-year re-revision rate for PS implants was 12,83% and 12,99% for semi-constrained, while the 10-years re-revision rate was 17,47% for PS implants and 16,91% for semi-constrained ones. These data were in accord with what was reported on the NJR and gave further validation to the study, thanks to the elevated number of patients involved (22,287 revisions). A Korean meta-analysis [22] from 2019, examining 12 studies, evaluated intermediate (5 years) and long (10 years) periods of follow-up of two types of constraint, CCK and Hinged. From their work emerged that 87.4% of Hinged and 83.8% of CCK prostheses survived at an intermediate follow-up, while 75.0% of CCK implants and 81.3% of Hinged implants survived at a 10-year follow-up. These data were influenced by the fact that some included studies treating joint bone tumors, such as Farfalli [4] article, where half of the cohort (22 out of 50 total people) included tumor resections, achieving survival for semi-constrained at 5 years of 69% and 62% at 10-years, while for the constrained the results were 83% at 5 years and 52% at 10 years. Considering the presence of oncologic patients in some studies accounts for the reduced survival rates in these papers.

Cherian [2] wrote in 2016 a metanalysis containing several studies concerning survival rates of rTKRs, using different degrees of constraint (PS, CCK, Hinged) in a selected population with TKR aseptic loosening. Meijer [15] analyzed the use of PS implants in rTKR and obtained a 2-year survival rate of 92% and 5-year survival of 92% on a sample of 60 patients. Laskin and Ohnsonge's [10] study achieved a 96% survival of PS implants used in rTKR at 5-years of follow-up. Mabry [14] in a retrospective study on PS implants in rTKR procedures obtained a survival at 5 and 10 years of 98% and 92%, respectively. Sappey-Marinier [18] investigate PS constraints used in primary and revision knee surgery, in selected cases of aseptic loosening, and reported no differences in clinical outcomes and survival rates (96,5% vs 100% at 3 years). These data show better results when compared to those obtained by RIPO. This is probably because septic loosening presents a worse prognosis than aseptic loosening, so the difference was justified by the fact that they had a selected study population, while our work included all cases. For CCK implants, one of the larger studies was by Wilke [21], with a cohort of 234 patients, where the survival rate, considering all causes of failure, was 91% at 5 years and 81% at 10 years. Another study by Luque [12] on 125 patients with CCK implants obtained a survival rate at 2 years of 92.7%, at 5 years of 87.8%, and at 8 years it remained constant. For hinged implants Gudnason [5] evaluated aseptic loosening on 42 prostheses with a 10-year follow-up: the survival rate obtained over the observed period was 89.2%.

Houfani et al. [7] reported in a retrospective study of 127 patients, treated with hinged implants for aseptic loosening, major instability, and mechanical failure, with overall survival at 5 years of 77%. Furthermore, the clinical outcomes evaluated showed improvement in different scores like in the total IKS score (+ 42 points), the IKS function score (+ 12 points), and the knee IKS score (+ 30 points).

Revision TKR does not necessarily need to use a higher degree of constraint, but an accurate patient selection is mandatory to obtain good results. From the analysis of the literature emerged that the difference in survival rate was more related to the type of cause that led to revision than to the degree of constraint, for example, aseptic loosening presented a longer survival when compared to septic, as demonstrated by the fact that if all pathological conditions that lead to the revision were included, the survivals reported in the literature were comparable to those of RIPO.

In conclusion, TKR failures can be managed with good results with different degrees of constraint, but usually it is common to choose a higher constraint than primary surgery. In our study emerged that CCK constraints are the most used in revision surgery and present an overall survival rate of 87.5% at 10 years of follow-up. Nevertheless, both higher and lower degrees of constraint can be safely used to manage rTKR with comparable results and should therefore remain a valid option when possible.
